# Potato Patatin Generates Short-Chain Fatty Acids from Milk Fat that Contribute to Flavour Development in Cheese Ripening

**DOI:** 10.1007/s12010-015-1569-3

**Published:** 2015-03-27

**Authors:** Robin E. J. Spelbrink, Hellen Lensing, Maarten R. Egmond, Marco L. F. Giuseppin

**Affiliations:** 1 AVEBE UA, Foxhol, The Netherlands; 2 AVERIS Seeds BV, Valthermond, The Netherlands; 3 Department of Membrane Enzymology, Utrecht University, Utrecht, The Netherlands

**Keywords:** Patatin, Food enzyme, Cheese lipase, Cheese flavour, Thermal inactivation kinetics

## Abstract

The potato lipase, patatin, has long been thought of as essentially inactive towards triacylglycerols. Recently, technology has been developed to isolate potato proteins in native form as food ingredients at industrial scale. Characterisation of native patatin obtained in this way revealed that this enzyme activity towards triacylglycerols has been underestimated. This enables the application of patatin in cheese ripening, which is described in this study. When patatin is added to milk during cheese making, the lipase preferentially releases short-chain fatty acids that contribute to cheese flavour in a dose-dependent manner. Fortuitously, the lipase activity is found mainly in the curd. The release of the short-chain fatty acids matches the activity profile of patatin towards homotriacylglycerols of defined chain length. Residual patatin in the whey fraction can be inactivated effectively by heat treatment that follows Arrhenius kinetics. The results are discussed in terms of cheese making, patatin substrate preference and implications for the use of patatin more generally in food emulsions.

## Introduction

The flavour of mature cheese is the result of a series of complex biochemical events, mainly produced by enzymes that occur in the curd during ripening. Among them, lipolysis of milk fat is carried out by lipolytic enzymes, which are hydrolases that cleave the ester linkage between a fatty acid and the glycerol core of the triacylglyceride, producing free fatty acids (FFA), and mono- and diacylglycerides. The principal sources of lipolytic agents in cheese are lipoprotein lipase of milk, lipases from acid lactic bacteria and non-starter acid lactic bacteria (NSLAB), moulds and yeasts, lipases from rennet paste and exogenous lipase [1, 2]

The use of exogenous lipases in dairy industry is growing not only as cheese flavour enhancer but also as an alternative to accelerate cheese ripening, a slow and relatively expensive process. Several preparations of lipases and esterases are commercially available, which are derived from moulds (*Aspergillus* sp., *Rhizomucor miehei, Pseudomonas fluorescens, Penicillium* sp.), from yeasts (*Candida rugosa)* and from the abomase of small ruminants. Lamb rennet paste containing pregastric esterase is used in the production of some PDO traditional sheep milk cheeses, such as Idiazabal and Roncal in Spain, Fiore Sardo, Pecorino Romano and Canestrato Pugliese in Italy and Feta in Greece [3–5].

Selecting an appropriate lipase and applying it in a specific food system can be difficult, since commercial lipases vary in activity over a range of six orders of magnitude. These preparations are often complex mixtures of materials that originate from the host organism. The presence of non-lipase enzymes can introduce undesired catalytic activity, while non-proteinaceous material can interfere with the control of water activity and with mass transfer processes [6]. Furthermore, addition of lipases in milk can pose an economic disadvantage when only a small fraction is retained in the curd [7, 8].

The potato tuber contains substantial amounts of protein. Large-scale production of such protein has been investigated by many authors using a variety of techniques [9]. One of the most convenient methods that have been identified thus far is the isolation of the major tuber storage protein patatin via chromatography [10].

Patatin is a glycoprotein that migrated to 45 kDa on SDS-PAGE gels and that exists in different isoforms that are immunologically identical [11]. It is ubiquitous among different potato cultivars and generally forms between 20 and 50 % of the potato tuber’s soluble proteome. While its physiological role is that of a storage protein, patatin shows hydrolase activity towards a broad range of substrate such as glycol- and phospholipids and their corresponding lyso-species, monoacylglycerols and methyl- and ethyl esters of fatty acids.

When hydrolyzing lipids, patatin shows a preference for the *sn*-1 position [12]. On paranitrophenyl substrates, patatin shows highest activity towards acyl chains of 8, 10, 12, or 16 carbon atoms [13]. Most authors agree that patatin has no activity towards triacylglyerols. Only recently, studies have suggested that this activity has been underestimated [14].

Currently, different native potato protein fractions are commercially available, e.g. food structuring based on the proteins functional properties [15], as well as for the fining of wine [16]. Surprisingly, patatin is recovered in a single fraction of high purity from potato juice by industrial chromatography commercially.

Some research was conducted on the practical use of patatin as a lipase for the synthesis of monoacylglycerols [17], but no commercial preparations would become available until the late 2000s. Since currently patatin is available in the native state at food grade purity, it is worthwhile to investigate its use in food processes such as cheese making.

Since the literature suggests a preference for specific fatty acids, namely capric and lauric acid, patatin is expected to preferentially release these fatty acids from cheese fat triglycerides [18, 13]. Specific fatty acids have distinct flavours that range from sharp/pungent for short-chain fatty acids to soapy/rancid/waxy or even bitter for the longer chain fatty acids [19]. A specific release of certain fatty acids can therefore confer a unique flavour profile to a cheese. In addition, these fatty acids can undergo subsequent conversion into other flavour components such as aldehydes, ketones and esters.

To evaluate the effect of patatin on cheese fat during ripening, Gouda-type cheeses were manufactured and analysed by chemical instrumentation and a trained test panel. Additional biochemical tests were performed to gain insight into the effect of patatin during cheese making.

For commercial application, cheese lipases should have minimal influence on the flavour and appearance of the resulting whey fraction. Hence, the distribution of patatin over the whey and curd fractions, and the effect of pasteurisation of the whey on residual lipase activity were determined.

## Experimentals

### Isolation of Patatin from Potato Tubers

Patatin was produced on the industrial scale according to the method in Giuseppin et al. [20]. Potato fruit juice from the AVEBE factory at Gasselternijveen, The Netherlands, was adjusted to pH 4.8. Five bed volumes were loaded onto an expanding bed adsorption column loaded with 40 cm of a custom agarose resin cross-linked with epichlorohydrin and supported on tungsten carbide beads that was modified with a mercaptobenzoic ligand (Upfront Chromatography, Denmark) at a flowrate of 5 cm/min. The column was washed with 1 bed volume of 30 mM citrate buffer at pH 4.8 and eluted with 5 bed volumes of 30 mM citrate at pH 6.0. The eluate was adjusted to a pH of 6.3 and concentrated to 10 % (*w*/*v*) protein on 30 kDa MWCO ultrafiltration membranes. Protein content was determined using a Sprint Rapid Protein Analyser (CEM) that was calibrated against Kjeldahl nitrogen using a conversion factor of 6.25. The resulting material was dried on a spray drying tower operating on at a temperature of 200 °C, resulting in an off-white powder. This material is sold commercially under the brandname “Solanic”. Lipase activity was determined according to the lipase activity method in the Food Chemicals Codex [21]. Briefly, this method uses a pH-stat setup to determine the amount of sodium hydroxide that is required to maintain the pH of an emulsion of tributyrin in gum Arabic in the presence of a lipase.

### Determination of Hydrolytic Activity of Patatin Towards Defined Substrates

Triacetin, tributyrin, tricaproin, tricaprylin and trilaurin were purchased as a standard kit from Sigma Aldrich (Tri11-1kt); 100 mM Stock emulsions of the substrates were prepared in reaction buffer. Tricaprin and trimyristin failed to yield stable emulsions and were omitted from the study. Substrates were added for a final concentration of 1 mM to 0.5 % (*w*/*v*) Gum Arabic (Sigma Aldrich) containing 100 mM Triton X100, 50 mM NaCl, 10 mM CaCl_2_ and 5 mM Tris(hydroxymethyl)aminomethane at pH 7.8.

Saturated phophatidylcholines of chain lengths of 6, 7, 8, 9 and 12 carbon atoms were purchased from Avanti Polar Lipids Inc. and hydrated in reaction buffer.

Patatin was added in quantities sufficient to induce a linear and consistent consumption of sodium hydroxide solution to maintain the pH. This consumption was measured over time and expressed as micromoles per minute per gramme of patatin.

### Distribution of Patatin Over Curd and Whey Via Lipase Activity Measurement

Lipase substrates were purchased from Sigma Aldrich (4-nitrophenylcaprylate, 21742); 5-mL aliquots of milk were supplemented with doses of patatin of 150, 250, 350, and 500 mg/L from a 100-g/L patatin solution. Total volume was kept constant between the different samples by the addition of demi-water. The resulting mixtures as well as untreated milk were coagulated by the addition of rennet (Sigma Aldrich R5876) at 10 mg/L and incubating for 90 min at 35 °C. The resulting material was separated into curd and whey by centrifugation at 9000×*g* for 10 min. The whey was then transferred into microvials and centrifuged again at 15,000×*g* for 10 min to obtain a slightly opaque solution. The curd fractions were resuspended in 100 mM citrate buffer at pH 7.5; 100 μL of these solutions were mixed with 100 μL of 30 mM Tris-HCl at pH 8.0 solution containing 2 mM of 4-nitrophenylcaprylate in a 96-well plate. The increase in absorbance at 405 nm was recorded at 10-s intervals for 5 min at ambient temperature using a BioRad Model 680 plate reader. For each sample, three data points were used. Since the presence of fat micelles strongly influenced the activity of patatin towards the lipophilic substrate, the activity of patatin in whey was compared with the activity of patatin in aqueous buffer and the activity of patatin in milk was compared with that activity in resuspended curd.

### Thermal Inactivation of Patatin

Patatin (Solanic 2x6P) was dissolved to a concentration of 1 g/L in buffer solutions of pH 5.0, 6.0 and 7.0 and in freshly prepared whey of pH 6.7. Kinetic degradation models of the lipase activity in these solutions were constructed by measuring residual activity upon thermal exposure in a stopped-flow system.

Whey was prepared by adding to whole milk 10 mg/L rennet at 30 °C for 90 min and removing the curd by filtration through cheese cloth. Patatin solutions were treated at temperatures between 50 and 80 °C at exposure times ranging between 4 ms up to 10 s. Lipase activity was determined by measuring the increase in absorbance at 340 nm of the patatin solution acting on 4-methylumbeliferyl acetate (Alfa Aesar A12147) in 30 mM phosphate buffer of pH 8.0 for 3 min [22]. The data were fitted according to Arrhenius kinetics according to the method of Anton and Barrett [23].

### Addition of Patatin to Model Cheeses

Model cheeses with patatin were manufactured and analysed at NIZO Food Research (Ede, The Netherlands) using the processing protocol for Gouda-type cheese in the Screencheese system which is described in detail in Bachmann et al. [24]. One vat of curd (200 L) was prepared as in usual cheese making, using thermised bactofugated standardised and pasteurised cheese milk. Rennet (Kalase, 150 IMCU, CSK Food Enrichment, Leeuwarden, The Netherlands) was added at a concentration of 230 μL/L milk and calcium chloride (33 wt.%) was added at 400 μL/L milk. Starter culture (Bos mesophilic starter, CSK Food Enrichment, Leeuwarden, The Netherlands) was pregrown for 20 h at 20 °C and added to the vat. This mixture was incubated for 45 min at 30.5 °C. The resulting curd was cut and washed at 30.5 °C by alternatingly cutting and stirring for 20 s and resting for 3 min, over a period of 20 min. The curd was then washed with water at 35.5 °C for 45 min. After washing the curd, it was divided into ten portions with different doses of patatin (Solanic 2x6P) in a 10 wt.% solution in milk mixed thoroughly through. These doses were 0.1, 0.5, 1, 3, 5, 10, 15, and 30 mg patatin/L cheese milk. Two portions were kept as untreated controls. The curds were pre-pressed, divided into four equal parts and each placed in a cheese vat to form an individual cheese. The resulting 40 cheeses (∼350 g each) were pressed, brined, vacuum-packed and ripened at 13 °C for 6 or 13 weeks.

### Volatile Compounds Analysis

Volatile flavour components were determined and quantified under the Screencheese procedure at NIZO Food Research (Ede, The Netherlands). This method consisted of solid-phase dynamic extraction of the headspace at 60 °C. The volatiles were then injected by a Combi Pal Autosampler directly into an Ultrafast GC-MS equipped with a UFM RTX 200, 10 m × 0.14 mm GC column that underwent a temperature gradient from 20 to 250 °C (Thermo Fisher Scientific). MS spectra were recorded over an *m*/*z* ratio of 35–350 by a Thermo Plus TOF MS (Thermo Fisher Scientific), operating in single-ion recording mode.

### Free Fatty Acid Analysis

Levels of individual fatty acids were determined in duplicate using GC-based methods as part of the Screencheese system at NIZO Food Research (Ede, The Netherlands) using the procedure of de Jong and Badings [25]. Briefly, to 1 g cheese samples, 3 g of Na_2_SO_4_, 0.3 mL of H_2_SO_4_ and internal standards are added, followed by three extractions in 3 mL of 1:1 ether/heptanes. Neutral lipids are separated from the FFAs by passing the pooled extracts over a 1-g deactived alumina column and eluting the neutral lipids with 10 mL 1:1 ether/heptanes. The FFA are then recovered by washing the alumina twice with 2.5 mL diethylether containing 3 vol.% formic acid; 0.5 μL of this extract is introduced directly into a Carlo Erba Model Mega 5160 GC (Carlo Erba, Milan, Italy) equipped with a fused silica capillary column and a flame ionisation detector. Samples were injected at 65 °C; during the run, the temperature was raised to 240 °C at 10 °C/min. Typically, the coefficient of variance for this analysis is less than 2 %.

### Sensory Analysis

Sensory analysis was carried out by a trained expert panel (*n* = 12) at NIZO Food Research (Ede, The Netherlands) that was trained and selected via an ISO 8586 procedure. The testers represent the 10 % best skilled individuals in smelling and tasting of the normal population and are regularly trained on dairy products and the Common Flavour Language (CFL). Cheeses were tested blind and in random order. The panel evaluated the attributes sweet, bitter, metallic, scorched, soapy/sweaty, farm-like and creamy.

## Results and Discussion

### Patatin Isolation

The patatin preparation obtained that was used in this research consisted mainly of protein (Table 1). Lipase activity was approximately 500 LU/g. This activity is on the low end of the scale among commercial lipase preparations [6]. In contrast, the purity in terms of protein content is among the highest.

**Table 1:** Composition of patatin preparation

Moisture	Protein	Activity
4–8 %	90 %	500 U/g

### Introduction of Patatin into Cheese

In cheese making, lipases can be introduced either in the milk fraction or directly into the curd prior to pressing. Patatin that is dissolved in milk fractionates mainly with the curd in enzymatic coagulation, as is common for lipases due to their affinity for fatty substrates. Approximately 30 % of the amount of patatin that is introduced remains in the whey fraction (Table 2).

**Table 2:** Distribution of patatin activity over the curd and whey fraction

Patatin concentration (g/L)	Added activity^a^	Activity in whey^b^	% of activity recovered in whey	Activity in milk^b^	Activity in curd^b^	% of activity recovered in curd
0.15	53	18.8 ± 0.2	35	20 ± 1.1	16.5 ± 1.2	83
0.25	89	27.9 ± 0.2	31	22 ± 3	16.8 ± 0.8	76
0.35	125	42.5 ± 0.3	34	30 ± 2	20.7 ± 1	68
0.50	178	62.2 ± 1.1	35	35 ± 2	24.8 ± 1.2	71

^a^Calculated from enzyme in buffer

^b^mOD405/min

### Effect of Patatin on Defined Lipids

**Fig. 1:**
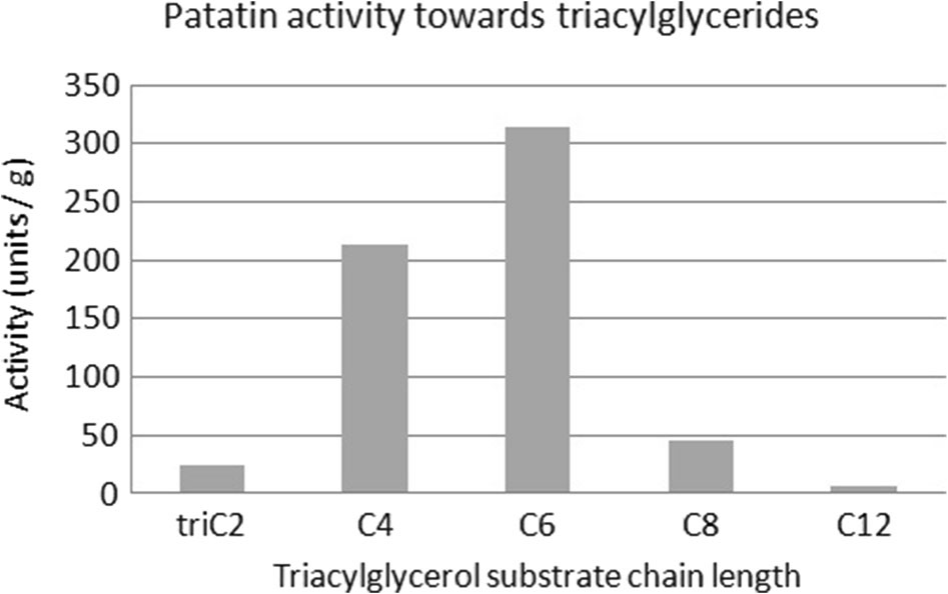
Activity of patatin towards triacylglycerides of defined acyl chain length determined via pH-stat

A series of experiments on defined substrates revealed that patatins activity towards triacylglycerides matches its preference for cheese fatty acids (Figs. 1 vs. 2). Interestingly, the preference of patatin towards homodiacyl-glycerophosphocholines (Fig. 3) is different from both the literature values obtained on paranitrophenyl esters and triacylglycerols alike [18, 13]. The hydrolytic activity of patatin towards phosphatidylcholine substrates is vastly higher than towards triacylglycerides, and the enzyme shows increased activity towards substrates of increasing chain length. However, when substrate chain length reaches a value of 12, activity is abolished.

**Fig. 2:**
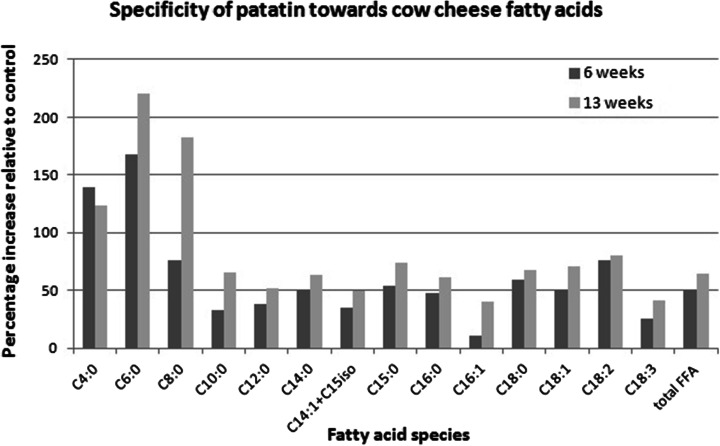
Selectivity of patatin towards different fatty acid species in Gouda-type cheese

**Fig. 3:**
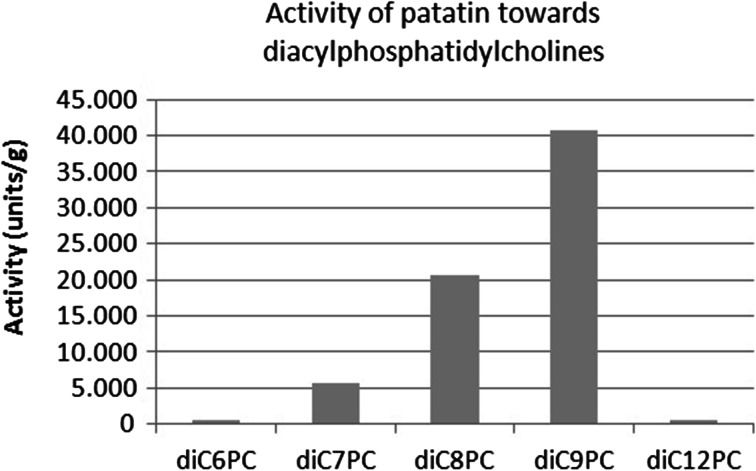
Activity of patatin towards diacylphosphocholines of defined acyl chain length determined via pH-stat

This lack of activity may be explained by the observations that posphatidylcholines of chain lengths of 12 and above tend to form stable bilayer structure in aqueous solution, while lower chain lengths form either leaky vesicles or micelles [26]. Lysophosphatidylcholines of any chain length also form micelles due to their conical molecular shape. From previous studies, it is known that patatin shows high activity towards lysophosphatidylcholines [13].

Overall, these results indicate that patatin is not selective for specific acyl chain lengths per se, but rather prefers to hydrolyze substrates that occur in a soluble or micellar form in aqueous solution. This behaviour may result from patatins lack of a hydrophobic lid [27]. This structure is commonly thought to mediate the association of lipases with hydrophobic surfaces and occurs in the vast majority of lipases that have been analysed to date.

This mechanism has implications for the application of patatin in cheese. Because patatin will only hydrolyse the most soluble, least hydrophobic triglycerides, the small, most pungent fatty acids will be exclusively released while the soapy larger fatty acids will remain in the triacylglycerides. Because the fraction of susceptible triglycerides will be small, the addition of patatin to cheese will only release a modest amount of free fatty acids. Thus, excessive lipolysis and rancidity could be avoided.

### Effect of Patatin on Cheese Lipids

**Fig. 4:**
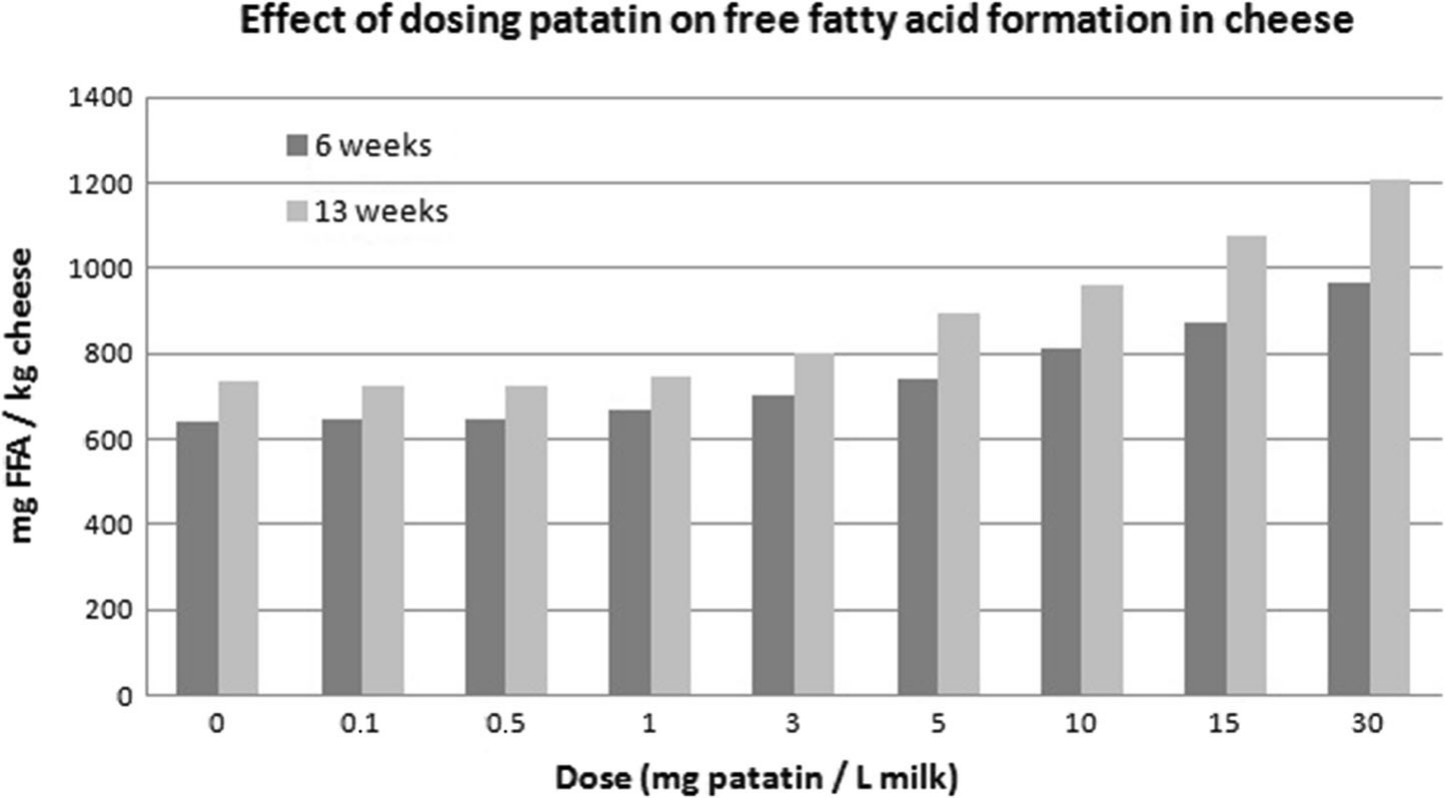
Effect of different doses of patatin on the release of free fatty acids in Gouda-type cheese after 6 and 13 weeks of ripening

The patatin-containing curds show increased levels of lipolysis compared with a control curd. Lipolysis increases in a dose-dependent manner with the amount of patatin that is introduced (Fig. 4). At a patatin dose of 1 mg/L milk, the free fatty acid content becomes statistically different from the controls. Surprisingly, the selectivity of patatin towards cheese fatty acids is different than would be expected based on the reported literature information. Instead of the expected preferential release of capric and lauric acid [13], the highest increase in fatty acids was observed for butyric, caproic and caprylic acid (Fig. 2).

### Effect of Patatin on Cheese Volatiles

**Fig. 5:**
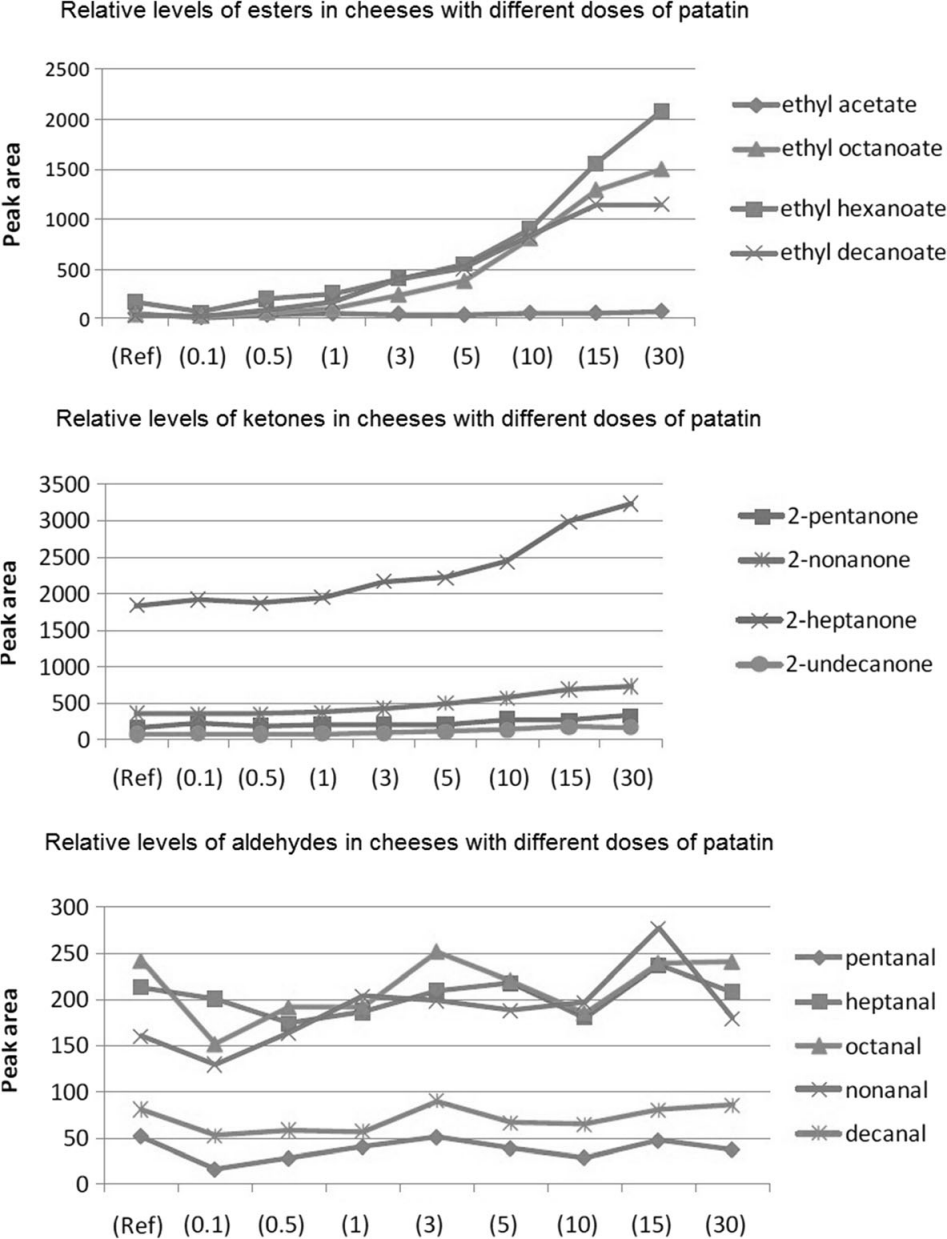
Increase in volatile components of cheese containing patatin after 13 weeks of ripening: *Top*, esters. *Centre*, ketones. *Bottom*, aldehydes. Patatin doses are expressed as milligrammes patatin per litre of milk

In addition to free fatty acids, a variety of derived products such as esters and methylketones were detected. The increase in esters could result from the direct action of patatin as a catalyst for ester synthesis. However, the increase could also result from non-patatin hydrolases that work on the released fatty acids. Fatty aldehydes did not increase in an appreciable manner upon the addition of patatin to cheese (Fig. 5).

### Effect of Patatin on Cheese Sensory Attributes

**Fig. 6:**
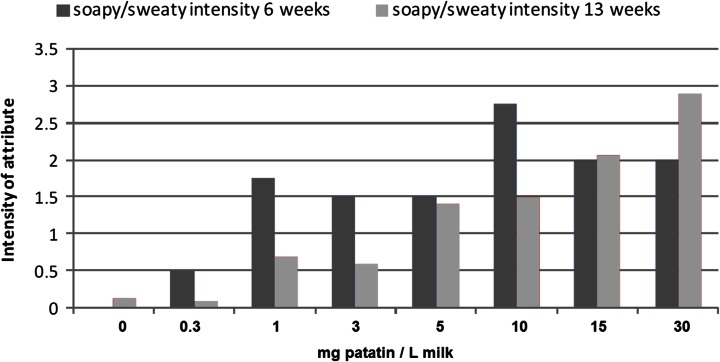
Increase in the soapy/sweaty flavour attribute of cheese containing patatin after 6 and 13 weeks of ripening

Several taste attributes were found to differ between the patatin cheeses and reference cheese: Bitter, sweet, scorched and soapy/sweaty. Of these attributes, only the soapy/sweaty flavour aspect varied in a consistent dose-dependent manner (Fig. 6). At a patatin dose of 0.5 mg/L milk, the test panel reported statistical difference with the reference cheeses. The increase in flavour is consistent with the increase in butyric, capric and caproic acid in the free fatty acid analysis. These flavours are most commonly associated with mould- and smear-ripened cheeses, blue cheese and hard-type Italian cheeses [19] like Romano, Pecorino, Provalone, Parmesan as well as Feta and Mozzarella.

While the levels of several volatile components increased in a dose-dependent manner with patatin, the typical flavours of these products were not reported by the test panel, the more pungent FFAs apparently dominate the flavour profile.

### Inactivation of Residual Patatin in Whey

Approximately 30 % of the patatin that is added to milk remains in the whey fraction, from which whey protein is recovered. The presence of enzymatically active material in this fraction could be highly undesirable. While the unfolding of patatin as a function of temperature has been reported previously as determined by far-UV circular dichroism [28] and differential scanning calorimetry [14], these data provide no insight into the kinetics of enzyme inactivation and therefore do not allow the effect of pasteurising conditions to be assessed accurately.

**Fig. 7:**
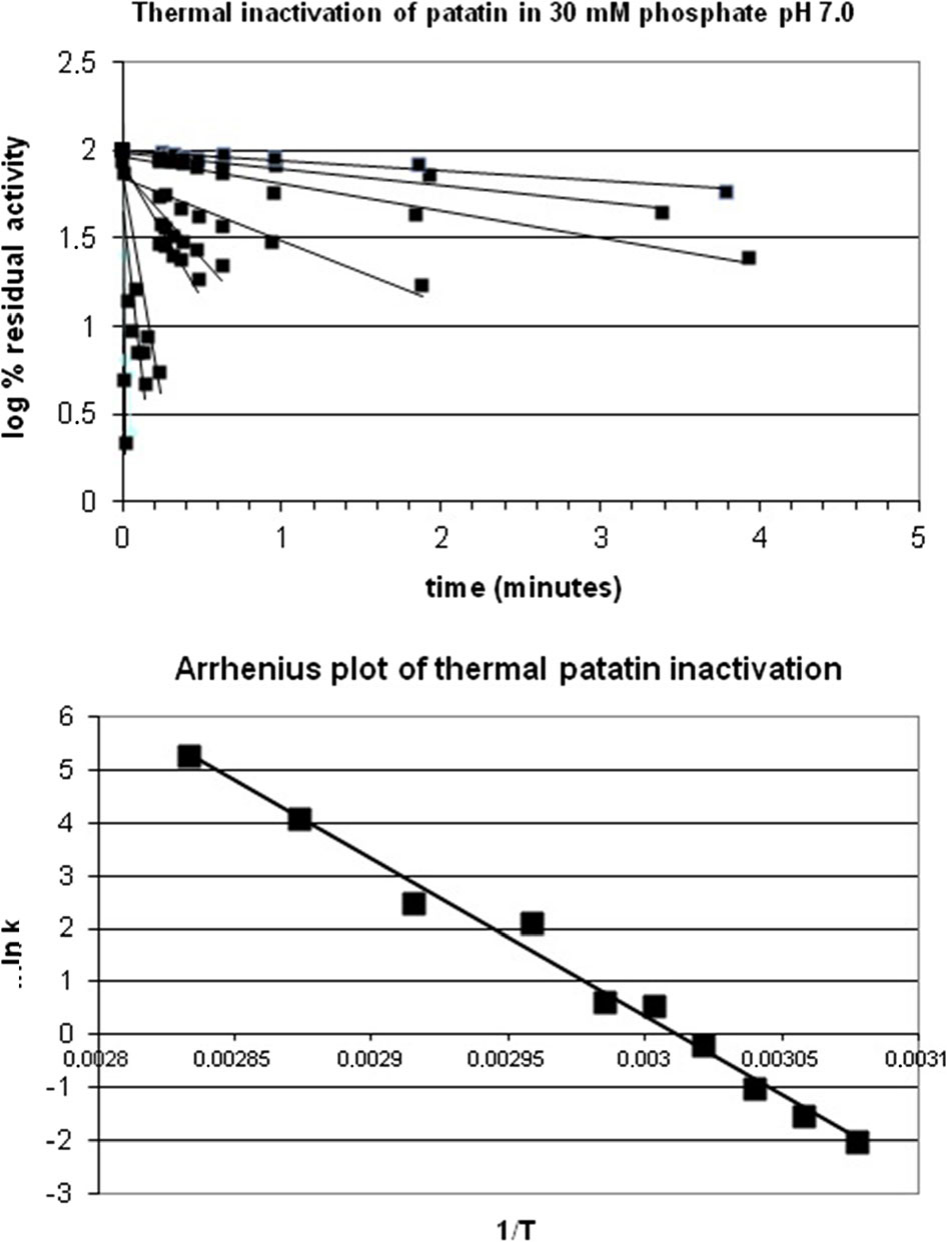
Thermal inactivation of patatin. Residual activity after heat treatment (*top*) and Arrhenius plot for the same (*bottom*)

To establish the required conditions for the inactivation of patatin, solutions of this enzyme were heat-treated in a stopped-flow system. This allowed for the treatment of patatin at defined temperatures and time intervals. Aliquots were analysed for residual lipase activity, and the inactivation characteristics were plotted essentially according to Anthon and Barrett (Fig. 7). Inactivation kinetics were recorded for patatin in buffer solutions of varying pH values and in whey solution. Typically, cheese whey undergoes a pasteurisation step at 74 °C. This temperature will substantially inactivate patatin in a matter of seconds (Table 3).

**Table 3:** Thermal inactivation parameters for patatin under different conditions

Inactivation conditions	Activation energy (kJ/mol)	Reference temperature (K)	reference reaction rate
30 mM phosphate pH 5	258 ± 15	333	0.53
30 mM phosphate pH 6	268 ± 14	333	0.57
30 mM phosphate pH 7	248 ± 10	333	0.56
Whey	205 ± 20	333	0.32

## Conclusions

The potato lipase patatin hydrolyses triacylglycerides that contain short-chain fatty acids in both model studies and in the context of ripening cheese. In cheese, fatty acids are released by patatin in a dose-dependent manner that causes a corresponding increase in the relevant flavour attributes. In addition, the presence of patatin in cheese results in an increase in esters and ketones but not in aldehydes. However, the typical flavours that are associated with these compounds were not detected by sensory analysis. Patatin fractionates mainly into the curd fraction in cheese making.

Patatin undergoes effective thermal inactivation at moderate temperatures according to Arrhenius kinetics in both aqueous buffers and in whey protein solution. This allows the use of patatin in cheese making for the development of cheese flavour without adverse effects on the whey protein fraction.

The use of an effective inactivation of the lipase by pasteurisation and the low levels of side reactions make potato lipase very suitable for controlled flavour formation derived from short-chain fatty acids in cheeses and other fermented products [29].

Next to cheeses, studies on model substrates reveal that patatin prefers to hydrolyse water-soluble compounds or compounds that are capable of forming micelles rather than continuous membranes or oil droplets. Hence, no lipolysis catalysed by patatin is expected for the majority of food fats and oils. This will enable lipase applications in emulsified food systems.

Previously, van Koningsveld and coworkers have proposed the use of patatin as an effective emulsifying agent in the preparation of food emulsions but raised concerns about residual lipase activity [14]. In the present study, no hydrolysis on long-chain triacylglycerides was observed. Since the vast majority of food fats and oils do not contain short-chain triacylglycerides (milk fat and to a lesser degree coconut oil being the exceptions), such hydrolysis is not expected to occur in food systems where native potato protein comes into contact with food fats and oils. This is in agreement with studies on the use of potato protein for the emulsification of soy oil [30] and corn oil [31]. While these authors did not specifically investigate lipolysis by potato protein, the nature of their experiments is such that it would have been revealed if it had occurred to an appreciable extent. However, the addition of susceptible triacylglycerides to such emulsions could result in the formation of free fatty acids in situ should such a system be desired to, for example, generate cheese-like flavour in emulsions.
